# The role of anti-citrullinated protein antibody in pathogenesis of RA

**DOI:** 10.1007/s10238-024-01359-3

**Published:** 2024-07-08

**Authors:** Hang Ma, Xu Liang, Shan-Shan Li, Wei Li, Tian-Fang Li

**Affiliations:** 1 Department of Rheumatology, The First Affiliated Hospital of Zhengzhou University, Zhengzhou, 450052 Henan China; 2 Academy of Medical Sciences, Zhengzhou University, Zhengzhou, 450052 Henan China

**Keywords:** Anti-citrullinated protein antibody, Peptidyl arginine deiminase 4, Rheumatoid arthritis, Neutrophil extracellular traps, Citrullination

## Abstract

Rheumatoid arthritis (RA) is a common autoimmune rheumatic disease that causes chronic synovitis, bone erosion, and joint destruction. The autoantigens in RA include a wide array of posttranslational modified proteins, such as citrullinated proteins catalyzed by peptidyl arginine deiminase4a. Pathogenic anti-citrullinated protein antibodies (ACPAs) directed against a variety of citrullinated epitopes are abundant both in plasma and synovial fluid of RA patients. ACPAs play an important role in the onset and progression of RA. Intensive and extensive studies are being conducted to unveil the mechanisms of RA pathogenesis and evaluate the efficacy of some investigative drugs. In this review, we focus on the formation and pathogenic function of ACPAs.

## Introduction

RA is a systemic autoimmune disease characterized by synovial inflammation and cartilage degradation. Lacking effective and prompt treatments may eventually lead to complete joint destruction, at which time, total joint arthroplasty is the only treatment of choice. The infiltration of immune cells in the synovial membrane, particularly, macrophages and lymphocytes, plays a central role in the pathogenesis of RA [1]. The generation of autoantibodies against a wide range of proteins including rheumatoid factor (RF), ACPA, anti-carbamylated protein antibody (anti-CarP), anti-acetylated protein antibody, etc., may be the causative factors for the initiation of RA [2]. Among them, ACPAs have demonstrated high specificity for RA diagnosis and a close association with disease activity [3]. Although RA is a highly heterogeneous disease, it can be roughly divided into two subtypes: ACPA-positive and ACPA-negative RA. Significant differences have been found between these two subtypes with regard to genetic background, environmental risk factors, disease progression, and remission [4]. Emerging data demonstrate that ACPAs can be detected many years before the onset of clinical RA and correlate with preclinical inflammation, the severity of joint disease, and increased radiographic progression [5]. Posttranslational modification (PTM) of proteins, particularly, protein citrullination mediated by PAD, is critical for the generation of the antigens that induce the formation of ACPAs [6].

Because of their particular importance in RA pathogenesis, more studies are needed to unveil the mechanisms for the early development of ACPAs and their diagnostic and prognostic values in pre-RA and clinical RA. Improved understanding of the formation of ACPAs may help the identification of novel and effective therapeutics for RA. In this review, we summarized recent progress in these fields and explored future research directions.

## The formation of ACPAs

It has been well accepted that uncontrolled activation of PAD4 and subsequent protein citrullination are critical for ACPA formation. We expect that further studies on the origin of ACPA formation during inflammation may lend novel insights into the initiation, formation, progress, prognosis, and potential identification of novel drugs for RA [7].

### Hyperactivation of PAD4

The PAD family members are Ca^2+^-dependent isozymes and share 50% sequence similarity [8]. To date, at least five subtypes of PADs have been identified in mammals, namely PAD1, PAD2, PAD3, PAD4/5, and PAD6 [9]. The distribution of the five PAD isoforms varies across tissues and organs. Among them, PAD4 is highly tissue-specific and abundant in the bone marrow and immune cells, and particularly, in neutrophils [10]. This enzyme can catalyze the deamination of arginine residues to produce the citrulline protein target of ACPA [11].

Membranolytic damage can increase Ca^2+^ influx onto target cells, and subsequently, cause the hyperactivation of PAD4 [12]. For example, the formation of neutrophil extracellular traps (NETs) is extruded from the cell and forms extracellular fibers that bind pathogens, causing membranolytic damage [13]. In addition, the membrane attack complex (MAC) formed by the complement system, perforin, and granzyme B released from cytotoxic cells can also induce membranolytic damage [14]. Among these, Ca^2+^ is a critical regulator for the catalytic activity of PAD4 as it can induce the structural change of PAD4, e.g., from the active site cleft, which causes the transition of an inactive to an active PAD4 conformation [15].

### Breach of self-tolerance

In the case of defect tolerance and prone genetic background, the disorder of PAD and atypical exposure, PAD4 can induce the formation of abnormal citrullinated proteins or peptides, triggering antigen-specific immune reactions in the genetically susceptible individuals to produce autoantibodies [16].

Genetic risk factors have been attributed to single nucleotide polymorphisms (SNPs) in a range of genes. The shared epitope (SE) in the MHC-II locus is an important risk factor as it contains the alleles that increase the risk of developing seropositive RA evidenced by epidemiological studies [17]. Some studies have clarified the important role of HLA-DRB1*01, HLA-DRB1*04 SE alleles and two non-SE HLA-DRB1 alleles (DRB1*13 and DRB*15) [18]. T cells can recognize citrullinated antigens in the context of HLA-DRB1*04, and the autoimmune B cell response encompasses a large spectrum of citrullinated proteins. It has been suggested that S2 and S3P, S1 and S3D alleles may also confer susceptibility factors to ACPA-seropositive RA [19, 20]. Genome-wide association studies (GWASs) have demonstrated at least 30 alleles associated with RA. Among those alleles, PTPN22, IL23R, TRAF1, STAT4, CD40, PADI4, IRF5, CCR6, and CTLA4 are of particular importance [21].

In addition to a genetic disposition, triggers at mucosal sites are thought to play a key role in these early events. When stimulated by some factors such as smoking, environmental dust, and microorganisms in periodontitis, macrophages, and other PAD-producing cells in the mucosa will be activated, resulting in the production of PAD [22, 23]. As accumulating data show that smoking and dust are associated with RA pathogenesis, it is widely accepted that the lung is the initial site of RA development [24, 25]. *Porphyromonas gingivalis* (*P. gingivalis*) and *Aggregatibacte*r *actinomycetemcomitans* (*Aa*) are crucial factors for periodontitis[26], *P. gingivalis* expresses PAD and citrullinated enolase, can mediate citrullination of bacterial and host protein. *Aa* hyperactivates PAD by inducing membranolytic damage on neutrophils[27]. These might participate in the breach of immune tolerance to PAD4 [28].

The PAD enzymes citrullinate a range of cytoplasmic, nuclear, membrane, and mitochondrial proteins. The dysregulation of PAD activity can drive the formation of abnormal citrullinated proteins or peptides which are exposed to the immune system, leading to the generation of citrulline-specific antibodies in a complex inflammatory environment such as the RA joint [29].

### The interaction in NETs, PAD4, and ACPA

The intracellular and extracellular activation of PAD4 may induce the citrullination of various proteins, including enolase, fibrinogen, vimentin, collagen, histone, etc.[30]. Upon PAD4 activation, locally released citrullinated histones enhance the generation of highly mutated clonal B cells resulting in the generation of high-affinity ACPAs [31].

Neutrophils are innate immune cells that may incite RA development when the immune tolerance is broken [32]. Neutrophil activation can lead to the extrusion of cellular DNA and protein complexes that form NETs with antimicrobial properties, through a form of cell death coined NETosis. NETs can enhance the immune response by capturing and killing bacteria [33]. However, they are indiscriminate in terms of cytotoxicity, and uncontrolled formation of NETs can damage healthy tissues [34]. The subsequent increase in Ca2^+^ influx on target cells may cause uncontrolled citrullination and loss of specificity. The aforementioned citrullination of nuclear histones by PAD4 is a trigger for the formation of NETs [35]. Neutrophils generate citrullinated epitopes and release peptidylarginine deiminase (PAD) enzymes capable of citrullinating extracellular proteins in the rheumatic joint, contributing to renewed ACPA generation [36]. As such, the protein motifs that are not citrullinated under healthy conditions may become citrullinated as new epitopes are generated. These new epitopes may then be recognized by the immune system as antigens and trigger antibody reactions. Therefore, locally released citrullinated histones enhance the generation of highly mutated clonal B cells resulting in the generation of high-affinity ACPAs [37].

**Fig. 1:**
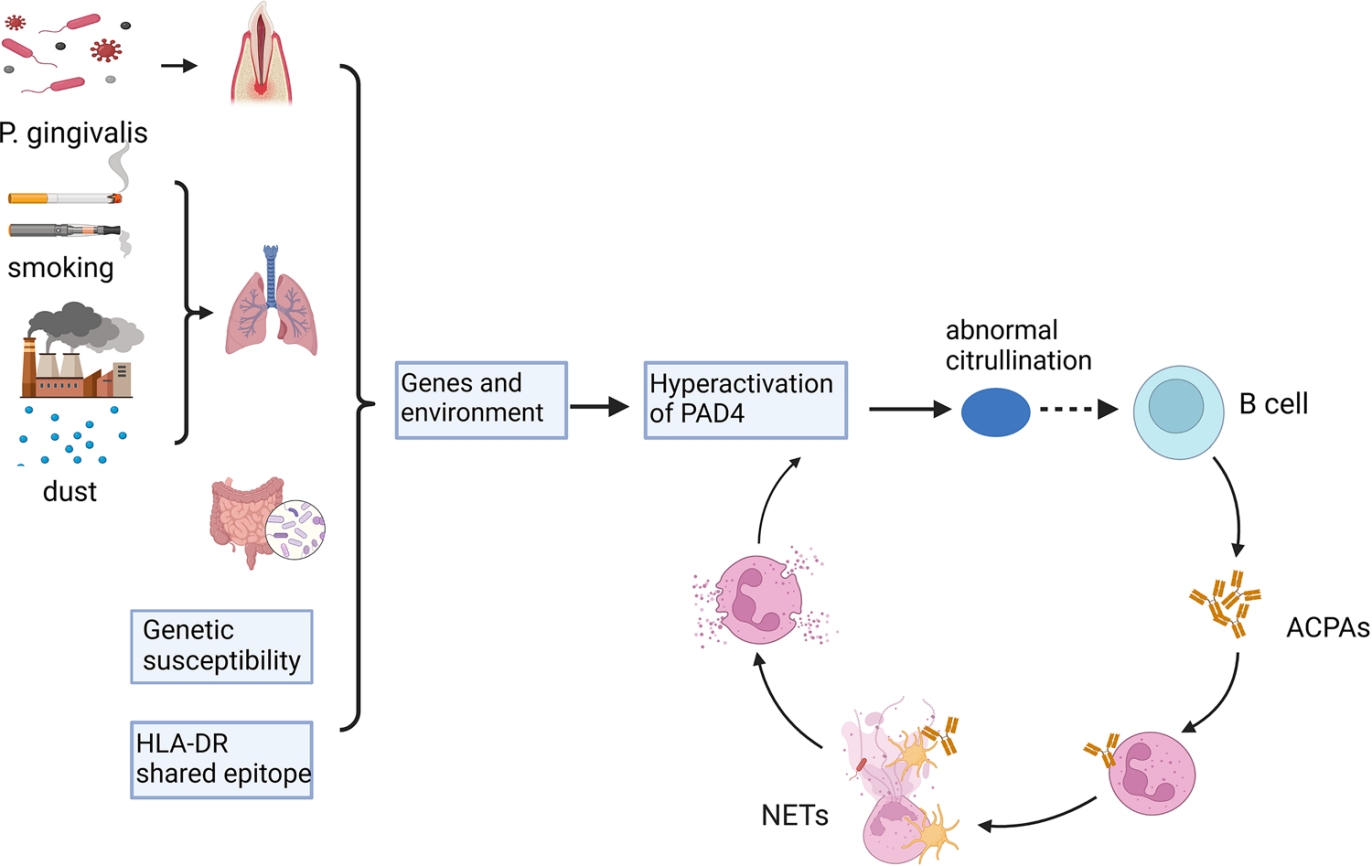
The activation of PAD4 and the interaction in NETs, PAD4 and ACPAs. Smoking, environmental dust, microorganisms in periodontitis, macrophages, and other PAD-producing cells in the mucosa may enhance the production of PAD4. With defective immune tolerance and genetic susceptibility, PAD4 can induce the formation of abnormal citrullinated proteins or peptides, promote the interaction of citrullinated proteins and the immune system, and cause genetically susceptible individuals to produce autoantibodies. Neutrophils are activated by immune complexes and inflammatory cytokines within the synovial fluid, frequently causing enhanced NETs formation in RA. In turn, NETs serve as a source of citrullinated autoantigens, further triggering the production of ACPAs. *PAD4* peptidylarginine deiminase 4; *ACPAs* anti-citrullinated protein antibodies; *NETs* neutrophil extracellular traps

Neutrophils are activated by immune complexes and inflammatory cytokines within the synovial fluid, frequently causing enhanced NET formation in RA [31]. ACPAs can also promote the release of PADs from neutrophils, which in turn catalyze the modification of arginine to citrulline, creating a vicious circle of autoantibody production [38, 39] (Fig. 1).

### Clinical relevance of autoantibodies in RA

**Fig. 2:**
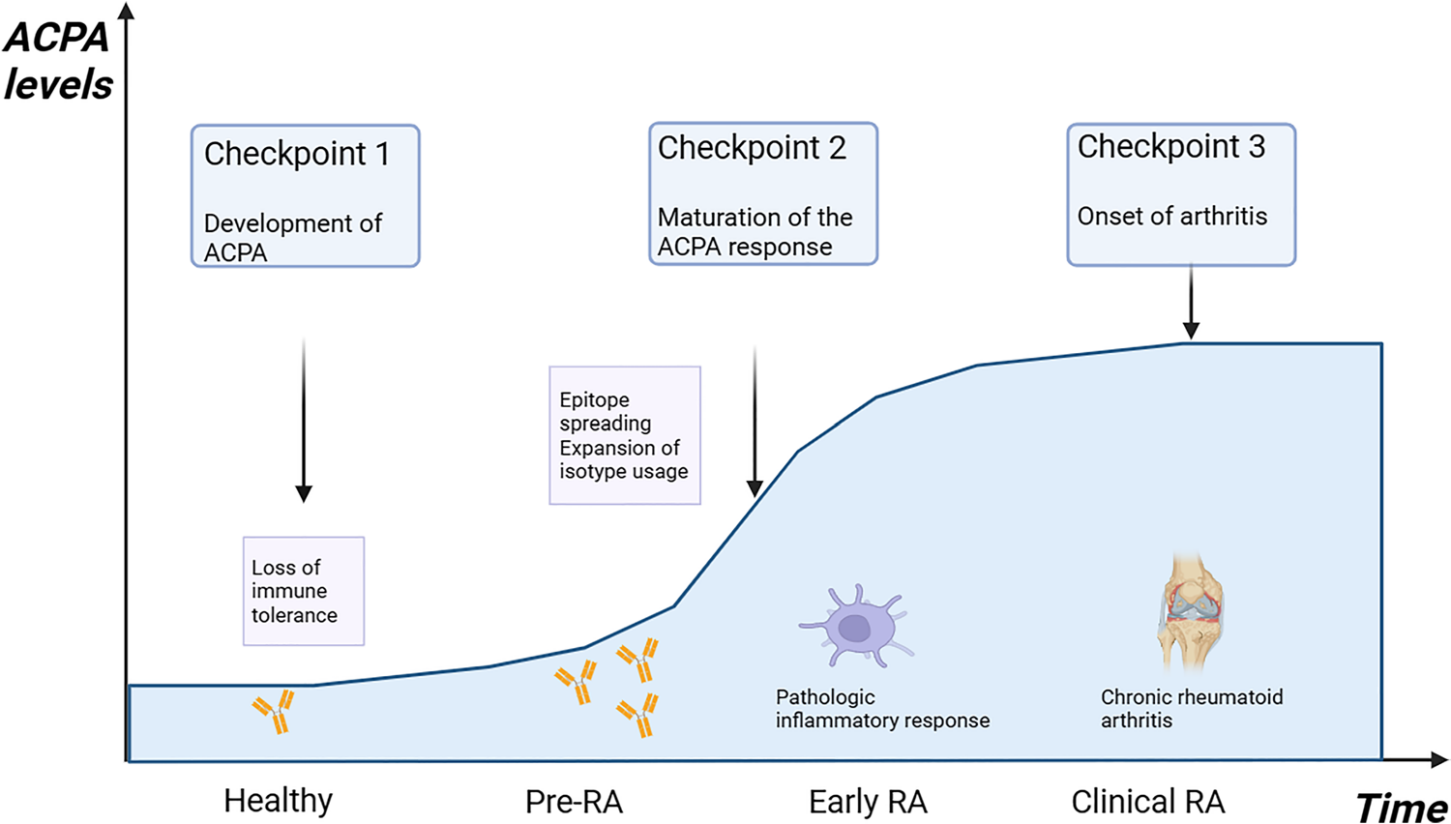
The development of autoimmunity and progression of RA. The prediction models suggest a cascade of autoantibodies, inflammation, and arthritis. While the specificity of autoantibodies binding to ACPA is confined and their isotype is limited in healthy individuals, epitope spreading and isotype expansion may occur in RA patients

RA is gradually developed from pre-RA, early RA to clinical RA with overt autoimmunity [40]. The immunopathogenesis of RA begins with the production of autoantibodies against post-translationally modified proteins, which by itself is initially reversible and self-limiting. (Checkpoint 1). After years of asymptomatic autoimmunity and progressive remodeling of the immune system, tissue tolerance erodes, and protective joint-resident macrophages fail, the ACPA response matures and accumulates more variable domain glycosylation sites (Checkpoint 2). Acute synovitis converts into chronic-destructive synovitis (Checkpoint 3)[36] (Fig. 2).

## Pathogenic effects of ACPAs

**Fig. 3:**
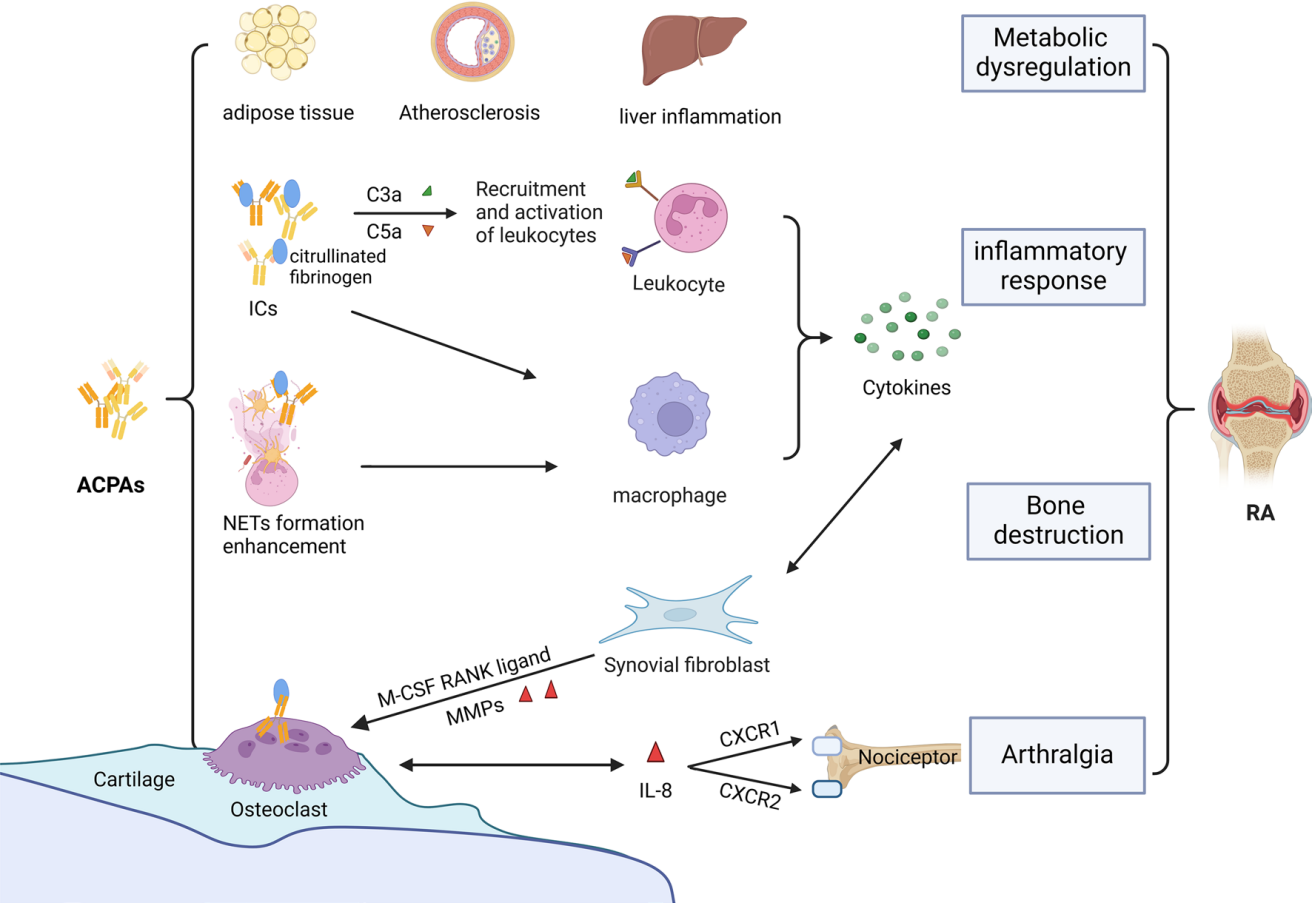
The pathogenesis of ACPAs in RA. Interaction between ACPAs and citrullinated fibrinogen forms ICs, which activate inflammatory cells and the complement system with subsequent release of C3a and C5a. The uncontrolled production of pro-inflammatory cytokines and mediators creates a local inflammatory milieu. These cytokines induce the generation of MMPs and RANKL by fibroblasts. While RANKL is closely involved in the formation and activation of osteoclasts causing excessive bone resorption, MMPs, particularly, MMP13, enhance cartilage degradation. The combined effects of these mediators eventually lead to complete joint destruction. ACPAs bind to osteoclasts, stimulating the release of IL-8 and autocrine enhancement of osteoclast maturation and activation. Further, chemokines such as CXCR1 and CXCR2 overexpressed in the sensory neurons may induce allodynia. ACPAs have the multi-faceted role of altered metabolites in adipose tissue, vascular, and liver tissue. *ACPAs* anti-citrullinated protein antibodies; *ICs* immune complexes; *NETs* neutrophil extracellular traps; *TLR-4* Toll-like receptor 4; *IL-1R* interleukin 6 receptor; *IL-6R* interleukin 6 receptor; *M-CSF* macrophage colony-stimulating factor; *RANK* receptor activator of NK-κB; *MMPs* matrix metalloproteinases; *IL-8* interleukin-8; *CXCR1* CXC chemokine receptor1; *CXCR2* CXC chemokine receptor; *RA* rheumatoid arthritis

As mentioned above, the ACPAs are of critical importance at the developmental stages of RA and are closely linked to both genetic background and the course of disease [41]. Mechanistic study indicates a direct link between the presence of ACPAs and bone erosions as well as pain in RA patients [42]. Central pathophysiological changes include synovial inflammation, cartilage destruction, bone erosion, and systemic inflammation [43]. In conclusion, an improved understanding of the role of autoantibodies in RA pathogenesis may facilitate the identification of novel therapeutics [44, 45] (Fig. 3).

### Activation of the inflammatory response

How might ACPA lead to inflammation? This could be mediated via binding to Fc receptors, NET formation or complement activation, which is described in more detail below.

#### Macrophage activation

Macrophages are important regulators of chronic synovial inflammation in RA [46]. Activated macrophages make up to 30–40% of the cellular content of the RA synovium. ACPAs can activate macrophages and promote the production of proinflammatory cytokines through an immune complex (IC)-mediated mechanism dependent on the Fcγ receptors and activation of the TLR4-MyD88 pathway [47]. Besides, ACPAs upregulate the interaction between CD147 and integrin-β1 in macrophages, which activates the Akt/NF-κB signaling pathway and upregulates the expression of NLRP3 and IL-1β. Further, a significant association between IL-23 and ACPA positivity in patients with untreated early RA has been reported [48, 49].

Activated macrophages in synovium recruit immune cells and fibroblast‑like synoviocytes (FLSs) by producing IL-1β, IL-6, TNF-α, IL-12, and other cytokines to promote inflammation [50], while chronic inflammation and cytokines secreted by other cells assist in the activation, polarization, and apoptosis of macrophages [51].

#### Neutrophils

ACPA ICs are present in the RA joint and can induce neutrophil degranulation by signaling through Fcγ receptors (FcγRs). ICs are potent pro-inflammatory stimuli of neutrophils that bind Fc receptors and trigger effector functions, including reactive oxygen species (ROS) production, degranulation, NETosis, and the generation of chemokines and cytokines [52]. Studies showed that ACPAs induce a defect in the miRNA biogenesis machinery in neutrophils from RA patients [53].

ACPAs promote NET formation, and this propagates a vicious cycle of inflammation, recruitment of leukocytes, and release of cytokines [54]. NETs externalize citrullinated antigens and enhance inflammatory response by inducing the expression of cytokines, chemokines, and adhesion molecules on synovial fibroblasts [55]. Their internalization by synovial fibroblasts or macrophages induces the release of various inflammatory cytokines.

#### Complements activation

Previous studies have demonstrated that ACPAs can recruit complements via both classical and alternative pathways. These studies suggest that ACPA-containing IC can induce inflammation in RA. They may enhance the immune response in RA by both FcγR binding and complement activation [56]. ICs can activate the complement system and induce the release of C3a and C5a, which then causes the recruitment and activation of leukocytes and the production of cytokines and other pro-inflammatory mediators [57].

### Bone destruction

Bone erosion is a cardinal sign of RA, and develops early after the onset of joint inflammation. A correlation between bone erosion and high levels of ACPAs has been reported [58]. Protein citrullination and ACPA binding to immature dendritic cells (DCs) might thus promote differentiation plasticity toward the osteoclast lineage, enhancing bone erosion adjacent to joints in ACPA-positive RA patients [59].

The presence of citrullinated proteins on the surface of osteoclast cells incites the binding of ACPAs to these cells and stimulates the release of IL-8, which facilitates the maturation and activation of osteoclasts via an autocrine mechanism [60]. In addition, ICs may further enhance osteoclast activation by engaging Fc receptors on their surface [61]. Macrophages induce the proliferation and activation of FLS by producing IL-1β and TNF-α. Activated FLSs secret RANKL and M-CSF and induce the formation and activation of osteoclast. Osteoclast formation can also be induced by L-1β, IL-6, and TNF-α produced by macrophages [62].

Interestingly, ACPAs can directly induce the differentiation of osteoclasts by binding the citrullinated vimentin on the surface of osteoclasts and mononuclear macrophage precursors [58]. Osteoclasts are derived from monocytes from the peripheral circulation. The process of differentiation for osteoclasts is mainly regulated by M-CSF and RANKL [63, 64]. Cytokines induce the production of MMP and RANKL by fibroblasts. RANKL activates osteoclasts and MMP causes tissue degradation, eventually to total joint destruction. With the accumulation of different ACPAs in joints, synovial inflammation occurs and increased production of cytokines and matrix-degrading enzymes ensues, leading to bone erosion and systemic osteoporosis in RA.

### Arthralgia

An experimental study demonstrated that the injection of ACPA into mice may significantly reduce the pain threshold. The attachment of ACPAs onto osteoclasts, these cells release IL-8 with subsequent recruitment of chemokines such as CXCR1 and CXCR2 to sensory neurons, causing allodynia [65, 66].

### Metabolic dysregulation

ACPAs have the multifaceted role of altered metabolites in the pathogenesis of RA. Arias-de la Rosa et al. have demonstrated that ACPAs may directly affect visceral human adipose tissue (AT) by regulating the genes related to inflammation, impaired insulin signaling, and alteration in lipid metabolism [67]. Previous studies have shown that the expressions of adipokines and adiponectin are dysregulated in RA patients, and such aberrant expressions of genes such as leptin are associated with the levels of ACPAs and the disease activity, indicating that AT may play an important role in autoimmunity and inflammation [68–72]. Adipokines may be involved in the pathogenesis of RA by breaking the integrity of the extracellular matrix in cartilage, dysregulating bone metabolism, modulating the immune system, and enhancing synovial angiogenesis [73].

Of note, ACPAs may incite vascular inflammation and coronary artery calcification by the yet-unknown mechanisms, which may at least in part explain the increased cardiovascular events in RA patients compared to healthy individuals [74–76]. ACPAs induced a defective hepatocyte function, promoting inflammation, apoptotic, and fibrotic processes [77]. Improved understanding of the correlation between different metabolites and disease severity may lend novel insights and facilitate the identification of new biomarkers and therapeutic targets for RA. The specific mechanisms of ACPA on inflammation, bone destruction, arthralgia, and metabolic disorders are summarized in Fig. 3.

## Strategies for interfering with ACPAs generation

Despite intensive research on RA drugs, some patients cannot achieve complete remission, and relapses may occur in those who have already achieved complete remission [78]. Thus, optimal therapies that can be widely applied to RA patients are currently unavailable. Such an unmet medical need can only be solved by an improved understanding of the pathogenesis of RA [78, 79]. Our review focuses on the generation and causative effect of ACPAs on RA as previous studies have shown that they are RA cascade leading to joint destruction. Early and effective intervention may ameliorate the severity of RA, which helps preserve the structural integrity of joints, and improve the patients’ quality of life [80] (Table 1).

### PAD4 inhibitor

PAD enzymes, particularly, PAD4, are critical for abnormal citrullination in RA, which can initiate and transmit autoimmunity of citrulline-related antigens, thus playing a unique role as an effector and target of autoimmunity reaction [81]. The in-depth understanding of the role of PAD enzymes in RA pathogenesis has led to the exploration of small molecules able to inhibit PAD activity [82]. PAD inhibition may block NF-κB signaling pathway and attenuate TLR-induced expression of IL-1β and TNF-α by neutrophils [83, 84].

Cl-amidine, an irreversible broad-spectrum PAD inhibitor via the modification of Cys645, may prevent the formation of NETs and alleviate joint symptoms in a mouse model of collage induced arthritis (CIA) [85], whereas it cannot inhibit osteoporosis in mice [86]. A preclinical study shows that BB-Cl amidine may alleviate immune-mediated arthritis in mice [87]. However, other reversible PAD4 inhibitors such as GSK199 and GSK484 have not been approved for clinical trials [88]. GSK199 inhibits the citrullination of PAD4 target proteins and diminishes the formation of NETs in vivo, and GSK484 inhibits H3-citrullination [83]. As an orally available inhibitor of protein arginine deiminase 4, JBI-589 is reportedly to ameliorate the damage caused by PAD4 and NETosis in mouse arthritis models [89]. Despite the discouraging results, the number of novel inhibitors keeps increasing, and some of which have shown excellent efficacy regarding reduced production of citrullinated proteins and relevant ACPAs in animal studies [90].

### NETs inhibitor

NETs have been implicated in many disease processes. Although they can eliminate pathogens, simultaneous tissue damage may occur due to the release of enzymes and other molecules [91]. Excessive formation of NETs is correlated to the severity of the disease, leading to tissue destruction and severe organ dysfunctions. Therapeutic ACPA (tACPA) may reduce the release of NETs from human neutrophils and enhance NET uptake by macrophages in vivo, thereby reducing joint damage and CIA progression in mice [92]. Targeting microRNA-155(miR-155) might be useful to inhibit exaggerated NET generation in inflammatory diseases [93]. Inhibition of TNF-α and IL-6 can reduce the formation of NET, which suppresses inflammation and serum markers, alleviates endothelial dysfunction, and inhibits immune cell activation [94].

### Targeting B cells

As B cell responses are necessary for autoantibody production, they naturally become the intervention target for RA treatment, particularly, in the early stage of disease progression. Deletion of B cells with simultaneous reduction in the levels of autoantibodies can abrogate the deleterious effects of these antibodies [95]. Targeting T cells using Abatacept causes significant decreases in the proportion of B cells in the synovium and ACPA-specific switched memory B-cells in the blood serum of RA patients [96]. Consistently, co-culture experiments have shown that anti-FITC CAR-T cells can eliminate ACPA-specific B cells from RA patients via recognition of corresponding FITC-labeled citrullinated peptide epitopes [97]. Inhibition of IL-6 using tocilizumab reduces the serum ACPA titer of RA patients by increasing the ratio of post-switch memory B cells (IgD-CD27^+^)/mature naive B cells [98]. A previous report shows that targeting B cells with rituximab results in a decrease in rheumatoid factor and serum ACPA levels in RA patients [99], however, we should keep in mind that not all B cells in RA patients are pathogenic and precise deletion of autoreactive B cells may achieve optimal outcomes with minimal adverse effects [100].

**Table 1:** Novel therapeutics related to citrullination

Drug category	Name	Target	References
PAD inhibitor	Cl-amidine	Via the modification of Cys645	[101]
	BB-Cl-amidine	Pan-PAD inhibitor	[102]
	GSK199	Inhibits the citrullination of PAD4 target proteins and diminishes the formation of NETs in vivo	[103]
	GSK484	Inhibit H3-citrullination	[104]
	JBI589	A non-covalent PAD4 inhibitor with high PAD4 isoform selectivity	[89, 105]
NETs inhibitor	Target miR-155	Inhibit exaggerated NET generation	[106]
	PGE2	Inhibit NET release	[107]
	tACPA	Diminish NET release and enhance NET uptake by macrophages in vivo	[92]
Targeting B cells	Tocilizumab	Increase the ratio of post-switch memory B cells (IgD-CD27^+^)/mature naive B cells	[108]
	Abatacept	Target B-cells by reducing CD80/CD86 expression	[109]
	Anti-FITC CAR-T cell	Eliminate ACPA-specific B cells	[97]
	Rituximab	Chimeric anti-CD20 monoclonal antibody	[110]

## Conclusions

Despite extensive and intensive studies, the detailed mechanisms for the pathogenesis of RA remain incompletely understood. Our review focused on the expression and activity of PAD4 as it is a critical enzyme for the formation of ACPAs. ACPAs can be detected many years before RA onset, early and optimal interventions to block RA cascade remain an unmet medical need and warrant more studies. Improved understanding of the association between PAD4, ACPAs, and genetic and environmental factors may facilitate the development of novel, safe, and effective therapeutic targets for RA.

## Data Availability

The data are available from the corresponding author upon reasonable request.
